# Rest–activity rhythm as a clinical biomarker: what are the next steps?

**DOI:** 10.1016/S2666-7568(23)00065-X

**Published:** 2023-05

**Authors:** Leandro C Brito, Saurabh S Thosar

**Affiliations:** Oregon Institute of Occupational Health Sciences, Oregon Health & Science University, Portland, OR 97239-3098, USA; OHSU-PSU School of Public Health, School of Nursing, Knight Cardiovascular Institute, Oregon Health & Science University, Portland, OR 97239-3098, USA

The endogenous circadian system drives physiological changes in accordance with periodic environmental changes, enabling humans to anticipate and adapt to daily changes—such as in temperature, feeding or fasting, and activity–rest cycles.^1^ There is an association between a disrupted endogenous circadian system and the development of some endocrine, cardiovascular, and neurological diseases.^2^

Regular physical activity at sufficient intensity is recommended for its inverse association with reduced risk of chronic diseases and mortality.^3,4^ A review of small, single-centre studies in controlled populations showed that managing the timing of structured exercise training programmes can affect glucose homoeostasis and cardiovascular system control (eg, blood pressure and heart rate).^5^ Not only does the timing of structured exercise matter, but the timing of physical activity might also be important for optimal health maintenance and chronic disease prevention.^6^ However, the association between rest–activity rhythm amplitude throughout the day and health outcomes remains unclear.

In *The Lancet Healthy Longevity*, Hongliang Feng and colleagues^7^ examine the associations between accelerometer-measured rest–activity rhythm amplitude and health risk in the general population. The authors conducted a retrospective analysis of 92 614 individuals with valid wrist-worn accelerometer data from the UK Biobank dataset, followed-up for an median of 6.4 years (IQR 5.8–6.9), and found that decreased rest–activity rhythm amplitude was associated with poor health outcomes and might constitute an important modifiable risk factor. Additionally, low rest–activity rhythm and moderate-to-vigorous physical activity amplitude had a stronger association with incidence disease and disease-specific mortality than other well documented rest–activity parameters (ie, sleep quality and other physical activity indicators), and the associations were robust and not modified by age or sex.

Rest–activity rhythm amplitude is easy to measure objectively and could become a promising tool to identify individuals at risk of adverse health outcomes in public health and clinical practice. Ascoli-Israel and colleagues^8^ have previously used relative amplitude to calculate rest–activity rhythm and suggested that rest–activity rhythm amplitude might help to minimise the misinterpretation of findings due to disturbances caused by poor sleep, reinforcing the robustness of this indicator. Feng and colleagues^7^ provided an important example of this association: an individual with sleep disturbances but a high level of daytime physical activity would still have a high rest–activity rhythm amplitude. Even though the potential of physical activity to mitigate the harmful effects of poor sleep is by no means an established fact, this finding is in accordance with a previous cohort in which associations between cardiometabolic diseases and sleep loss and poor sleep quality occurred mainly in individuals with low physical activity levels.^9^

Despite its potential for widespread use and its importance for clinical practice and public health, the development of rest–activity rhythm amplitude as a clinical biomarker is still in the early stages. Among other limitations, the observational nature of the retrospective analysis does not allow cause–effect conclusions to be drawn; randomised clinical trials will be needed to investigate the longitudinal effects of rest–activity rhythm amplitude on health outcomes. Low diversity in the individuals in the study also limits the generalisability of the findings;^7^ thus, future studies should ensure that data is collected in low-income and middle-income countries where opportunities for structured exercise and built environments conducive to physical activity might be scarcer than in high-income countries.

Notwithstanding the limitations, including those noted by the authors, rest–activity rhythm amplitude has immense potential for future research, which is essential to establish the clinical meaning and relevance of this biomarker.
